# Potential Output, EU Fiscal Surveillance and the COVID-19 Shock

**DOI:** 10.1007/s10272-020-0895-z

**Published:** 2020-06-07

**Authors:** Philipp Heimberger

**Affiliations:** The Vienna Institute for International Economic Studies, Rahlgasse 3, 1060 Vienna, Austria

## Abstract

This paper discusses how the technical foundations of the EU’s fiscal rules constrain the fiscal space in EU countries in the context of the COVID-19 pandemic. We review the evidence on how estimates of potential output, which are at the heart of essential control indicators in EU fiscal surveillance, were revised in the ten years running up to the COVID-19 pandemic, and how these revisions affected the fiscal stance of EU countries. We provide first evidence for downward revisions in the European Commission’s potential output estimates against the background of the COVID-19 shock across the EU27 countries, and we assess the potential consequences in terms of fiscal space. According to our results, one additional percentage point in predicted losses of actual output is associated with a loss in potential output of about 0.6 percentage points. Given the importance of model-based estimates in the EU’s fiscal rules, avoiding pro-cyclical fiscal tightening will require that policymakers’ hands are not tied by overly pessimistic views on the development of potential output.

The economic repercussions of the COVID-19 pandemic across Europe are severe. The immediate response of national EU governments has been to put forward discretionary fiscal measures to mitigate the macroeconomic shock. Based on a recommendation by the European Commission (2020a), the European Council activated the general escape clause in the Stability and Growth Pact. While this step temporarily provides fiscal space for individual governments to run larger fiscal deficits, there remains the question about the coordination of fiscal policies in Europe once there is a decision to end the suspension of the fiscal rules.

This paper provides a first analysis on how the technical foundations of the EU's fiscal rules contribute to shaping the fiscal space of individual EU countries in the aftermath of the COVID19-shock. In this context, the European Commission's potential output (PO) model serves as the core technical backbone of EU fiscal surveillance (e.g. Costantini, 2017; Heimberger et al., 2019). The European Commission uses the PO model for estimating the 'output gap', i.e. the difference between actual output (GDP) and a model-based 'potential output'. The output gap is interpreted as an indicator for the cyclical position of an economy: a negative output gap signals underutilisation of resources, a positive output gap indicates 'overheating'. Output gap estimates provide strong guidance for the Commission's judgments on how much of the actual fiscal deficit in a respective EU country is 'structural' in the sense that it is neither attributable to the effects of business cycle swings on government spending and tax revenues nor to budgetary one-off effects (Mourre et al., 2014; Buti et al., 2019).

The Commission's model-based estimates are used for evaluating and supervising member states' fiscal performance and underlie the Commission's recommendations related to medium-term budgetary objectives in the Stability and Growth Pact and in the Fiscal Compact (European Commission, 2019). Previous research on European fiscal policy in the aftermath of the global financial crisis has shown that this setup implies that model-based estimates of the 'structural' deficit feed directly into fiscal policy: when the estimate of the structural deficit is high(er), the fiscal space in individual member states is (more) constrained, as the countries concerned are obliged to adapt to tighter fiscal constraints (Klär, 2013; Tereanu et al., 2014; Truger, 2015; Heimberger and Kapeller, 2017; Fatas, 2019). In what follows, we review the role of model-based estimates in the EU's fiscal regulatory framework. Based on a review of how the technical founda-

**Philipp Heimberger**, Vienna Institute for International Economic Studies; and Johannes Kepler University Linz, Austria.

tions of the existing regulatory framework have affected fiscal policy coordination across the EU countries in the ten years running up to the COVID-19 pandemic, we provide a first analysis on how revisions in the Commission's estimates of potential output and 'structural' balances in the context of the COVID-19 shock will contribute to shaping fiscal space in individual EU member countries once the fiscal rules are again activated.

## Model-based estimates in the EU's fiscal rules

Potential output, defined as the level of output in an economy at which all production factors are employed at 'non-inflationary levels', is a theoretical concept with no observable empirical counterpart. The European Commission uses a Cobb-Douglas production function to provide estimates for potential output (Havik et al., 2014). This PO model is the preferred operational surveillance tool when it comes to evaluating fiscal policies in EU countries. It supplies estimates of potential output, which translate into estimates of the structural fiscal balance ($SB_t$) by using the relative difference between actual output and potential output – the so-called output gap ($OG_t$), as shown in Figure 1.

The institutional relevance of these model-based estimates is rooted in the EU's fiscal regulatory framework: the Stability and Growth Pact defines EU countries' medium-term budgetary objectives (MTOs) in terms of the structural fiscal balance. In case of a deviation from the MTO, a country has to reduce 'excessive structural deficits' by correcting the structural balance by 0.5% of GDP per year (e.g. European Commission, 2019). The expenditure rule implies that growth in public expenditures must not exceed growth in potential output. Furthermore, the Fiscal Compact refers to estimates of the structural deficit by stipulating that the structural deficit must not exceed 0.5% of GDP per year – a rule which signatory states had to codify into national law, preferably as a constitutional safeguard (Treaty on Stability, Coordination and Governance, 2012). As a consequence, larger estimates of 'structural' deficits amplify the pressure to implement fiscal consolidation measures.

## Downward revisions in potential output in the aftermath of the global financial crisis

Existing research provides in-depth analysis of revisions in potential output estimates in the aftermath of the global financial crisis (Klär, 2013; Ball, 2014; Tereanu et al., 2014; Palumbo, 2015; Truger, 2015; Heimberger and Kapeller, 2017; Fatas, 2019). All these studies find evidence for systematic downward revisions in potential output across the EU's member countries over the ten years leading up to the COVID-19 pandemic.

Figure 1

**Structural fiscal balances based on output gap estimates**

$$SB_t = \underbrace{FB_t - \epsilon_t OG_t}_{\text{cyclically adjusted balance}} - OE_t$$

cyclically adjusted balance structural fiscal balance ($SB_t$); fiscal balance ($FB_t$); budgetary elasticity ($\epsilon_t$); output gap (estimate based on PO-model) ($OG_t$); budgetary one-off effects ($OE_t$)

Source: Heimberger and Kapeller (2017).

To empirically illustrate the extent of downward revisions in potential output, we use the methodology developed in Ball (2014) and extrapolate the developments in potential output estimates before the financial crisis in 2007 (PO**) to compare these pre-crisis trends with potential output estimates in Autumn 2019 (PO*), i.e. the most recent estimates before the start of the COVID-19 pandemic.[1] From the y-axis values in Figure 2, it can be seen that losses in potential output in the year 2019 – which are calculated relative to extrapolated pre-crisis trends – vary markedly across European countries, ranging from 47.6% in Greece and 30.0% in Spain to much smaller losses in countries such as Germany (0.4%). The x-axis values depict losses in actual output. It can be seen that the losses in actual output and potential output are almost perfectly correlated, suggesting that the countries most affected by the crisis suffered the largest downward revisions in potential output – and vice versa.

## How do potential output estimates translate into 'structural' deficits? The example of Italy

Via the institutionalisation of structural balances in the EU's fiscal regulation framework, downward revisions in potential output increased fiscal consolidation pressures especially in the countries with the largest downward revisions. Negative output gaps would have been much larger than the Commission's official numbers suggested if the underlying views on potential output had been less pessimistic. As a consequence, several EU countries would have reached their medium-term budgetary targets much earlier, which would have provided them with additional fiscal space considering the EU's fiscal rules.

1 The Commission's forecast from December 2007 provides time series data for potential output for all EU countries through 2009 (we exclude five countries for which the 2007 data could not be compared to the Autumn 2019 data). We take these pre-crisis data, denote them by PO**, and extend all time-series beyond 2009 by means of log-linear extrapolation. Specifically, we compute the average annual change in the logarithm of PO** during 2000-2009, and then assume that potential output has increased at a constant rate from 2010 to 2019 (see Ball, 2014, 150).

Figure 2

**Downward revisions in potential output in pre-COVID-19 times**

Correlation of actual and potential output losses, 2019

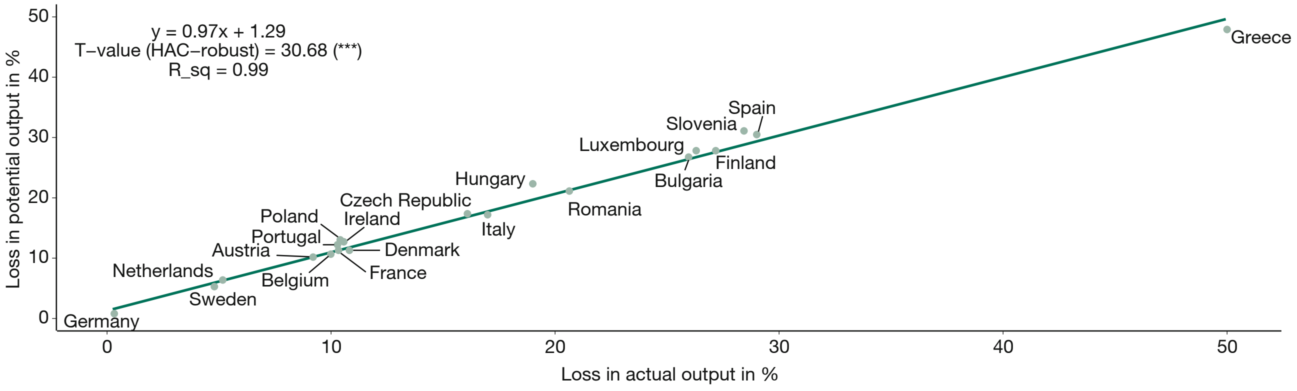

Note: Loss in potential output = (PO**-PO*)/PO**. Loss in actual output = (PO**-Y)/PO**. PO** denotes extrapolated estimate of pre-crisis PO (AMECO, Autumn 2007). See Ball (2014, 150) for details on the extrapolation methodology. PO*stands for PO estimate (AMECO, Autumn 2019). Y denotes real GDP (AMECO, Autumn 2019). *** denotes statistical significance at the 1% level. Six EU27 countries were excluded because their data from the Autumn 2007 forecast could not be compared to the Autumn 2019 data.

Source: AMECO (Autumn 2007, Autumn 2019); own calculations.

To illustrate this point, we use the example of Italy. The reason for this choice is that the Italian authorities were in open dispute over the Commission's estimates of potential output in pre-COVID-19 times. In June 2019, the European Commission recommended the opening of an excessive deficit procedure (EDP) because of violations of the EU's fiscal rules. Although the ultimate political decision was against opening a new EDP for Italy, the underlying technical debate remains unresolved. Italian authorities argued that the Commission was systematically underestimating the underutilisation of economic resources in the Italian economy, i.e. that the official estimate of the negative output gap based on the Commission's PO model was too small due to a pro-cyclical estimation bias. The Italian argument was that a correction of the Commission's estimates regarding the position of the Italian economy in the business cycle would drastically reduce requirements in terms of fiscal consolidation (Gualtieri, 2019).

According to the estimates derived from the PO model, Italy's economy was not suffering from underutilisation of economic resources in pre-COVID-19 times. In Autumn 2019, the output gap was estimated to stand at -0.2% for the year 2019, meaning that the Italian economy operated nearly fully in line with its potential output, despite the fact that the Italian unemployment rate still stood at around 10% and inflation was below 1%. Nonetheless, based on this output gap assessment, the European Commission's recommendations saw no fiscal space as the PO model's conceptual foundations suggested that expansionary fiscal policies would have risked overheating the Italian labour market (Heimberger, 2019).

Slow growth in the Italian economy over the ten years preceding the COVID-19 shock, however, had a strong impact on the Commission's potential output estimates. Figure 3 shows that before the start of the global financial crisis, the Commission estimated a steady growth trend in potential output. However, it then revised Italy's official potential output estimates downwards in several steps as the country's economic crisis deepened. According to the Autumn 2019 estimates, potential output still remains below the level reached before the global financial crisis.

In what follows, we apply the same approach as in Figure 2 to the Italian case, i.e. we extrapolate the pre-crisis developments in Italian potential output. In particular, we use the Commission's model-based potential output estimates produced back in 2007 (before the start of the financial and economic crisis) and extend them by using a constant growth trend for the years 2010-2019.[2] By using this simple trend extrapolation, we find a large negative output gap (the difference between actual output and potential output) of -16.9% of GDP for the year 2019, which starkly contrasts with the official Commission estimate of -0.2%. Whereas the European Commission's official potential output estimate in Autumn 2019 (i.e. before the start of the COVID-19 pandemic) bends down to meet actual GDP, the trend extrapolation shows a large and growing negative output gap, indicating underutilisation of economic resources (see Figure 4).

2 The extrapolation is based on the average potential output growth rates from the 2000-2009 period.

Figure 3

**Potential output model revisions for Italy**

Downward revisions in potential output over time

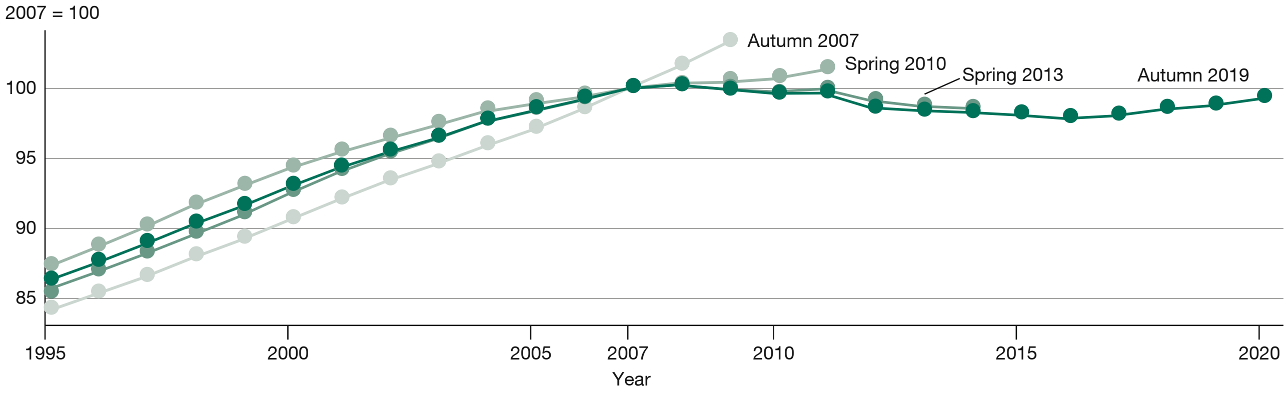

Source: European Commission forecasts (Autumn 2007; Spring 2010; Spring 2013; Autumn 2019); own calculations.

A simple trend extrapolation, however, is arguably problematic: downward revisions may be justified insofar as the crisis has triggered hysteresis effects. The concept of hysteresis postulates that inadequate demand during crisis times may have long-run effects on the supply-side potential of an economy, e.g. when long-term unemployment leads to skill losses among those who lost their jobs during the crisis (e.g. Ball, 2014; Blanchard et al., 2015).

To account for this hysteresis argument, we assume that the crisis indeed reduced the growth in potential output for the Italian economy. Over the period 2000-2009, the average growth rate of potential output was estimated to be 1.5% (based on the Commission estimates in Autumn 2007). Even when we assume that Italy's potential output growth rate was cut by two-thirds compared to the pre-crisis growth rate (making it 0.5% instead of 1.5%), which implies substantial hysteresis effects from 2010 onwards, the negative Italian output gap remains substantial (-8.5% of GDP).

We can demonstrate the relevance of different output gap estimates for Italy by looking at their implications for the fis-

Figure 4

**Alternative potential output estimates for Italy, 1999-2019**

At constant 2005 prices

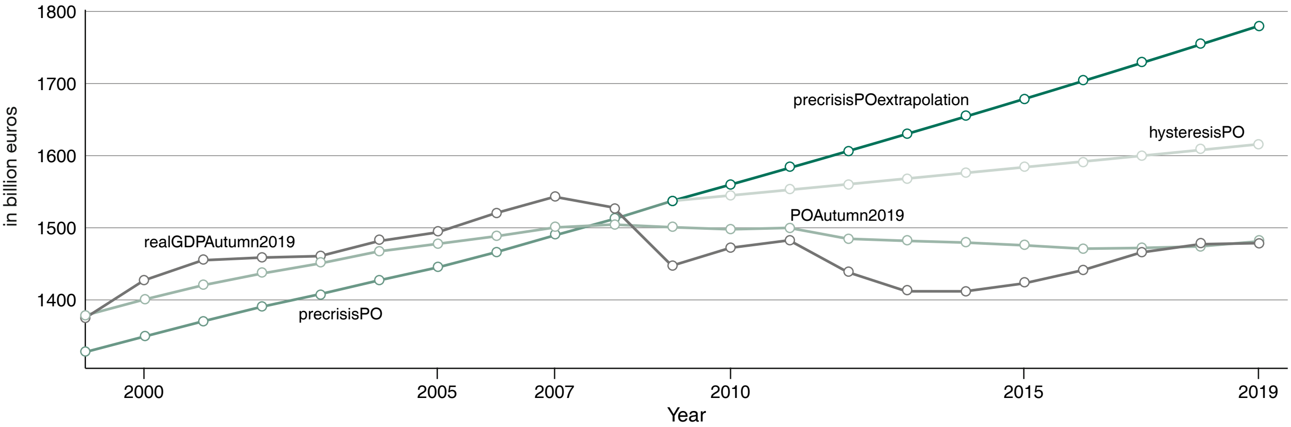

Note: precrisisPO denotes pre-financial-crisis potential output estimate (AMECO, Autumn 2007). POAutumn2019 stands for Commission's potential output estimates in Autumn 2019. precrisisPOextrapolation stands for extrapolation of the pre-crisis growth trend in potential output (see Ball, 2014, 150). realGDPAutumn2019 denotes real GDP in Autumn 2019. hysteresisPO stands for extrapolation of potential output based on the assumption that the pre-crisis growth in potential output has been cut by two-thirds.

Source: AMECO (Autumn 2007, Autumn 2019); own calculations.

cal space according to the EU's fiscal rules. Right before the coronavirus shock hit, the Commission estimated that the Italian fiscal deficit would come in at 2.2% of GDP in 2019. Given small official estimates of the output gap, the 'structural' deficit (2.2%) was estimated to be as large as the headline deficit. This model-based estimation implied that the Italian government would not meet its medium-term budgetary target, as this target does not allow the 'structural' deficit to exceed 0.5% of GDP. As a consequence, the Commission continued to demand 'corrective' fiscal consolidation measures in the years running up to the coronavirus pandemic.

However, Table 1 indicates that Italy would have been running a large 'structural' fiscal surplus of 6.9% of GDP in 2019 if we simply extrapolate the pre-financial crisis potential output growth rates (implying an output gap of -16.9% of GDP). Even under the hysteresis scenario, which accounts for the argument that post-crisis potential output growth was lower than in pre-financial crisis times (but not negative, as suggested by the Commission's official Autumn 2019 estimates), the 'structural' fiscal surplus in 2019 would have been 2.4% of GDP.

Therefore, alternative estimates of the output gap pointing to a higher degree of resource underutilisation would have reduced the fiscal consolidation pressure on the Italian government in pre-COVID-19 times. The Italian state would have overachieved its medium-term budgetary target, and the Commission's recommendation for lower government expenditure growth in the face of an officially small output gap would have been obsolete.

The European Commission has loosened rigidities related to the 'structural deficit' to some extent in 2014 by introducing so-called 'flexibility clauses' in the Stability and Growth Pact (European Commission, 2015). This step was at least partly a response to the criticism voiced by several EU member states over the course of the European debt crisis, where the critics included Italy, Spain, Latvia, Lithuania, Luxembourg, Portugal, Slovenia and Slovakia (Ciucci and Zoppe, 2016; Heimberger et al., 2019).

The flexibility guidelines establish a direct link between the size of the output gap and the required fiscal adjustment effort. In the case of a larger output gap (i.e. when the model estimates suggest that there is a lot of economic slack), little or no fiscal adjustment is required. The Commission's flexibility guidelines state that in "exceptionally bad times, interpreted as an output gap below minus 4% of GDP or when real GDP contracts, all Member States, irrespective of their public debt levels, would be temporarily exempted from making any fiscal effort" (European Commission, 2015, 21). But in the case of a small output gap, the fiscal

Table 1
**Alternative estimates of the output gap for Italy, 2019**

| | Potential output | Output gap | Fiscal balance | 'Structural' balance |
|---|---|---|---|---|
| Commission (official) | 1481.1 | -0.2 | -2.2 | -2.2 |
| S1: Pre-crisis trend | 1779.7 | -16.9 | -2.2 | 6.9 |
| S2: Hysteresis effects | 1615.8 | -8.5 | -2.2 | 2.4 |

Notes: Potential output in billion euros at constant 2005 prices. Output gap, fiscal balance and structural balance in percent of GDP.

Source: AMECO (Autumn 2019); own calculations.

consolidation requirements increase substantially. This step of introducing 'flexibility' provides additional leeway in the political case-by-case assessment. Paradoxically, however, it has further increased the relevance of the underlying estimates with the European Commission's model, and thus the importance of technical details. As a consequence, it would have mattered a great deal in the years prior to the COVID-19 shock if the negative output gap had been estimated to be larger than 4% of GDP in Italy and other countries, because the Commission's own guidelines would have pointed to the need to stop requirements for further fiscal consolidation.

While we used the example of Italy for illustration purposes, downward revisions in potential output closely related to actual output losses also systematically affected the fiscal space in other EU countries (see Figure 2). Especially in the period 2010-2014, the reliance of European fiscal policymakers on pessimistic views of potential output triggered procyclical adjustments in fiscal policy with negative economic growth effects (e.g. Truger, 2015; Heimberger and Kapeller, 2017). Fiscal consolidation caused hysteresis effects (Fatas and Summers, 2018), leading to successive rounds of downward revisions in potential output that partly validated the original pessimistic potential output forecasts and, in turn, caused further fiscal consolidation requirements (Fatas, 2019).

## First evidence on downward revisions in potential output in response to the COVID-19 shock

How will the COVID-19 shock affect the European Commission's potential output estimates, which are at the heart of EU fiscal surveillance? To provide a first analysis concerning the impact of the downturn in economic activity on estimates of potential output in the context of the coronavirus crisis, we compare the estimates from the Autumn 2019 forecast and

Figure 5

**Revisions in potential output due to the COVID-19 shock, 2021**

Correlation of actual and potential output losses: Spring 2020 forecast vs. Autumn 2019 forecast

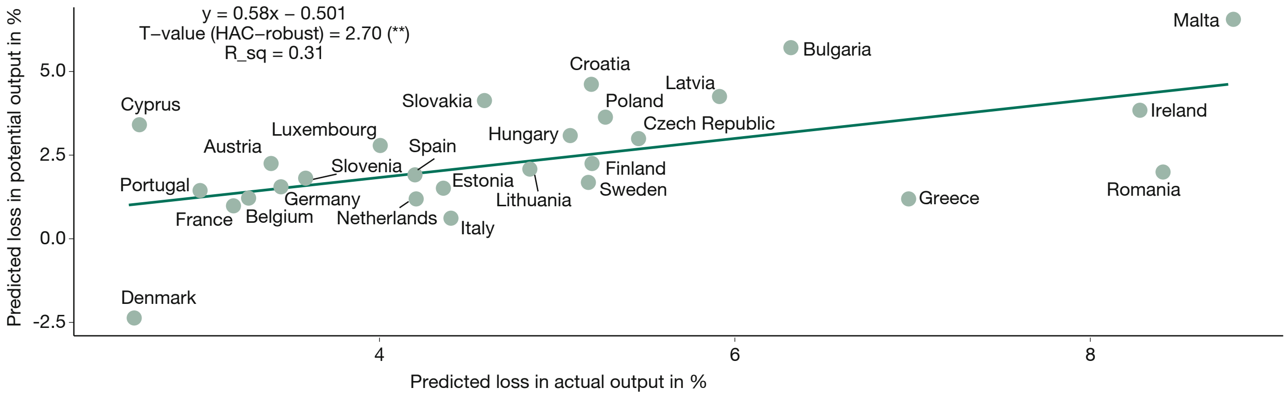

Notes: Loss in potential output = (PO**-PO*)/PO**. Loss in actual output = (PO**-Y)/PO**. PO** denotes potential output estimate (AMECO, Autumn 2019). PO* stands for potential output estimate (AMECO, Spring 2020). Y stands for real GDP (AMECO, Spring 2020).

Source: AMECO (Autumn 2019, Spring 2020); own calculations.

the Spring 2020 forecast, which provides the first estimates after the start of the pandemic.

While Figure 2 looked at data on actual GDP and potential output losses in the year 2019 relative to trends before the financial crisis, Figure 5 is based on the Commission's forecasts for the year 2021. We pose the question: what size are the revisions in actual and potential output when we compare the most recent pre-COVID-19 forecast with the Spring 2020 forecast. From the y-axis of Figure 5, it can be seen that estimated potential output losses for 2021 range from 6.4% in Malta to -2.5% in Denmark. In fact, Denmark is the only EU27 country that has not experienced a downward revision in potential output.

The regression line indicates a statistically significant positive relationship between potential output losses and actual output losses. In other words, the Commission systematically reduced its potential output forecast to a larger extent in countries that are also predicted to suffer from a larger drop in actual output. The correlation is not perfect, but one additional percentage point in predicted losses of actual output is associated with a loss in potential output of about 0.6 percentage points, and the simple bivariate regression explains more than 30% of the cross-country variation in estimated potential output losses. This first look at how the COVID-19 shock affects the Commission's potential output estimates provides evidence that the PO model continues to produce estimates that are systematically pro-cyclical in the sense that revisions in the PO model estimates are strongly related to changes in economic activity.

Downward revisions in potential output translate into higher 'structural' deficits, which will again become important once the suspension of the EU's fiscal rules is lifted. To illustrate this point, Table 2 compares official and alternative estimates for 'structural' fiscal balances in all EU27 countries.

The first column of Table 2 shows the official estimates of the 'structural' fiscal balance in the Commission's Spring 2020 forecast for the year 2021. The second column represents alternative estimates of the 'structural' balance, where we assume no downward revision in potential output compared to the Autumn 2019 forecast, i.e. potential output remains constant at the most recent pre-COVID-19 estimate. It can be seen that the estimated potential output losses (see Figure 5) lead to a more pessimistic view of the size of the 'structural' deficit across the EU27 countries, with the most pessimistic turn in countries with the largest downward revisions in potential output (see, e.g. Malta and Bulgaria).

Once the fiscal rules begin to take effect again, downward revisions will make it more difficult for several EU27 countries to meet their medium-term budgetary objectives. For illustration purposes, consider the example of Germany: in 2021, the downward revision in Germany's potential output implies a 'structural' deficit of 0.5% of GDP. However, the structural balance would be in surplus if we assume no shift to a more pessimistic view on potential output in comparison to pre-COVID-19 levels. References to the 'structural' deficit – estimated based on the Commission's PO model – are also at the heart of Germany's constitutional 'debt brake' (e.g. Hein and Truger, 2014). Once the clauses that exempt the 'struc-

Table 2
**Alternative estimates of 'structural' fiscal balances for the year 2021 in the EU27 countries**
Spring 2020 estimates vs. estimates without a downward revision in potential output compared to Autumn 2019

| Country | SB official | SB no revision |
|---|---|---|
| Austria | -1.14 | 0.06 |
| Belgium | -2.91 | -2.25 |
| Bulgaria | -1.62 | 0.03 |
| Croatia | -1.89 | 0.08 |
| Cyprus | -2.11 | -0.45 |
| Czech Republic | -2.91 | -1.81 |
| Denmark | 0.64 | -0.77 |
| Estonia | -1.90 | -1.25 |
| Germany | -0.50 | 0.20 |
| Finland | -1.59 | -0.41 |
| France | -2.46 | -1.93 |
| Greece | 0.81 | 1.33 |
| Hungary | -3.08 | -1.76 |
| Ireland | -0.45 | 1.40 |
| Italy | -3.70 | -3.44 |
| Latvia | -3.85 | -2.31 |
| Lithuania | -1.58 | -0.82 |
| Luxembourg | 0.69 | 1.90 |
| Malta | -1.34 | 1.66 |
| Netherlands | -1.57 | -0.95 |
| Poland | -3.10 | -1.38 |
| Portugal | -1.17 | -0.48 |
| Romania | -9.25 | -8.69 |
| Slovakia | -3.96 | -2.45 |
| Slovenia | -1.20 | -0.42 |
| Spain | -5.22 | -4.19 |
| Sweden | -0.17 | 0.65 |

Note: SB denotes structural balance in % of potential output.

Source: AMECO (Autumn 2019; Spring 2020); own calculations.

tural' deficit limits from being applied are lifted, a more pessimistic view concerning potential output will systematically restrict the fiscal space that is available to German policymakers when it comes to supporting the recovery.

## Conclusions

It is a Herculean task to provide a real-time assessment about how much of the output losses in the context of the COVID-19 shock will turn out to be permanent. The extent of hysteresis effects will to a large extent depend on the effectiveness of fiscal policy measures when it comes to mitigating the macroeconomic downturn and supporting economic recovery. A stronger recovery would imply limited and less persistent effects on potential output, but this outcome is contingent on allowing for properly expansionary policies as long as the recovery is incomplete. This paper has documented downward revisions in potential output by the European Commission across the EU27 countries, and these downward revisions tend to be stronger in those countries that are also forecast to suffer a larger decline in economic activity relative to the pre-COVID-19 levels. According to our results, one additional percentage point in predicted losses of actual output is associated with a loss in potential output of about 0.6 percentage points. The problem with downward revisions in potential output is that past research on the links between potential output and EU fiscal surveillance shows that pessimistic initial views have proven to be self-reinforcing as they reduce fiscal space exactly in those times when it is most needed (e.g. Truger, 2015; Heimberger and Kapeller, 2017; Fatas, 2019).

Policymakers have to expect that the views produced by the PO model will become more pessimistic in case of a deepening of the current economic crisis. Downward revisions in potential output imply relatively smaller output gap estimates, which (*ceteris paribus*), directly filter into larger 'structural' deficits. The EU's fiscal rules have been temporarily suspended in response to the outbreak of the coronavirus pandemic, but once this suspension is lifted, model-based assessments of excessive 'structural' deficits in the context of the EU's fiscal rules will force the countries concerned to implement fiscal consolidation measures that may hinder economic recovery. This may trigger a negative feedback loop, where restrictive fiscal policies accelerate the downturn in economic activity that partly validates the initial pessimistic view, leading to further rounds of downward revisions in potential output that systematically restrain the fiscal space for conducting anti-cyclical fiscal policy. Research has shown that pro-cyclical fiscal tightening has pronounced negative growth effects (e.g. Blanchard and Leigh, 2013; Jorda and Taylor, 2016), which aligns well with the finding that aggregate demand was squeezed the most in those European countries that implemented the harshest fiscal austerity measures during the years of the European debt crisis (De Grauwe and Ji, 2013; Heimberger, 2017; House et al., 2019).

Although the PO model has been revised in several steps over the last years (e.g. Heimberger et al., 2019) and the problem of pro-cyclical estimation biases is well known, the analysis presented in this paper suggests that the need for reforming the underlying estimation procedure is as pressing as never before. It is well known that real-time estimates of potential output are quite uncertain and revision-prone even in normal times. In the context of the COVID-19 pandemic, however, uncertainty is exceptionally high, and future fiscal policy should not be systematically restricted by highly revision-prone model estimates with a pro-cyclical bias. A pragmatic solution would therefore be to lock in the potential output estimates produced in Autumn 2019 (before the outbreak of the coronavirus pandemic) until a more reliable approach to estimating potential output has been developed. Such an approach would need to go beyond temporary adjustments for individual countries in the existing modelling framework.

An evaluation of the EU's fiscal rules and debates about technical reform options was already underway at the beginning of the year 2020, as the European Commission initiated a process of reviewing economic governance. In an accompanying document, the European Commission (2020b) argues that "the framework relies heavily on variables that are not directly observable and are frequently revised, such as the output gap and the structural balance, which hampers the provision of stable policy guidance" (10). Avoiding pro-cyclical policies in the months and years to come will require a reform of the PO model on which the cyclical adjustment of fiscal control indicators in the EU's fiscal rules is built. There are numerous technical papers that work towards achieving better real-time estimates, which are less prone to suffer from systematic pro-cyclical revisions (e.g. Coibion et al., 2018; Jarocinsky and Lenza, 2018; Fontanari et al., 2019).

Importantly, the European Commission's PO model would need to consider the presence of hysteresis effects. As noted by Fatas (2019), properly accounting for hysteresis "should make economic policies (fiscal and monetary) much more aggressive, in particular during large negative cyclical events like the one the euro area experienced during 2008-2014" (700). We already know that the COVID-19 crisis falls into the category of large negative cyclical events, and more flexibility will be required to avoid a vicious circle of feedback triggered by the application of the EU's fiscal rules in the near future, where downward revisions in potential output require additional fiscal consolidation measures, and fiscal consolidation undermines the recovery and eventually adversely affects public debt sustainability.
